# Supplementary material for: Retroactivity induced operating regime transition in an enzymatic futile cycle
Source: PLoS One. 2021 Apr 30;16(4):e0250830. doi: 10.1371/journal.pone.0250830 (PMC8087108; doi:10.1371/journal.pone.0250830)
Supplement: S1 Text — S1.1- Steady-state solution of the full model. S1.2- Dose-response curve transition from Signal-Transducing to Hyperbolic regime induced by substrate retroactivity. S1.3- Minimum retroactivity strength required to transition from one regime to another. S1.4- Effect of retroactivity strength on EC50 during different regime transitions. S1.5-Sensitivity and rate balance analysis to investigate retroactivity induced ST to H and TH to H regime transitions. (PDF) [file pone.0250830.s001.pdf]

<u>Text S1</u>

Retroactivity induced operating regime transition in an enzymatic futile cycle

Akshay Parundekar, Ganesh A Viswanathan*
Department of Chemical Engineering
Indian Institute of Technology Bombay
Powai, Mumbai - 400076
India
Ph: +91-22-2576-7222
*Email: ganeshav@iitb.ac.in

S1.1. Steady-state solution of the full model

The full ODE model of the single enzymatic cascade with retroactivity (Fig. 1, main text) corresponding to the biochemical reactions in Eqs 1-4 from Appendix I is

$$\frac{dms_1}{dt} = k_{on,1}m^u.s_1 - k_{off,1}ms_1 \quad [S1.1]$$

$$\frac{dm_ps_2}{dt} = k_{on,2}m_p^u.s_2 - k_{off,2}m_ps_2 \quad [S1.2]$$

$$\frac{d\,em}{dt} = k_1e.m^u - \left(k_{-1} + k_f\right)em \quad [S1.3]$$

$$\frac{d\,pm_p}{dt} = k_2p.m_p^u - (k_{-2} + k_r)pm_p \quad [S1.4]$$

$$\frac{dm_p}{dt} = k_fem + k_{-2}pm_p - k_2m_p^u(p_t - pm_p) \quad [S1.5]$$

along with the conservation relations

$$m = m^u + ms_1 \quad [S1.6]$$

$$m_p = m_p^u + m_ps_1 \quad [S1.7]$$

$$m_t = m + m_p \quad [S1.8]$$

$$e_t = e + em \quad [S1.9]$$

$$p_t = p + pm_p \quad [S1.10]$$

For obtaining the steady-state solution of the full model, we set the lhs to zero for the above five equations (Eqs [S1.1] - [S1.5]. Upon plugging in the conservation relation [S1.6] and [S1.7], respectively into Eqs [S1.1] and [S1.2], respectively with lhs set to zero, the concentration of the intermediate complex species MS_1 and M_pS_2 in the sequestration reactions is given by

$$ms_1 = \frac{k_{on}ms_1}{k_{off}+k_{on}s_1} = \frac{ms_1/K_d}{1+s_1/K_d} = \frac{m\lambda}{1+\lambda} \quad [S1.11]$$

$$m_ps_2 = \frac{k_{on}m_ps_2}{k_{off}+k_{on}s_2} = \frac{m_ps_2/K_d}{1+s_2/K_d} = \frac{m_p\alpha}{1+\alpha} \quad [S1.12]$$

where, $K_d = k_{off}/k_{on}$. Incorporating conservation relations Eq. [S1.6] to [S1.9] and the concentration of species MS_1 and M_pS_2 (Eqs S1.11 and S1.12) into Eqs [S1.3] and [S1.4], with lhs set to zero, concentration of the intermediate complex species can be written as

$$em = \frac{e_t m}{K_1(1+\lambda)+m} \qquad \text{[S1.13]}$$

$$pm_p = \frac{p_t m_p}{K_2(1+\alpha)+m_p}. \qquad \text{[S1.14]}$$

Using Eq. [S1.10], setting lhs to zero for Eq. [S1.4] also leads to the relation $k_2(p_t - m_p s_2).m_p^u = (k_{-2} + k_r)pm_p$, which when incorporated into the steady-state form of Eq. [S1.5] leads to

$$k_f em + k_{-2}pm_p - k_2 m_p^u(p_t - pm_p) = k_f em - k_r pm_p = 0 \qquad \text{[S1.15]}$$

Substituting Eqs [S1.13] and [S1.14] into [S1.15] leads to the relationship

$$\frac{k_f e_t(m_t - m_p)}{K_1(1+\lambda)+(m_t-m_p)} - \frac{k_r p_t m_p}{K_2(1+\alpha)+m_p} = 0. \qquad \text{[S1.16]}$$

This algebraic expression upon re-arranging leads to a quadratic equation, solving which one can arrive at the expression for m_p at steady-state given by

$$\bar{m} = \frac{m_p}{m_t} = \begin{cases} \frac{-b+\sqrt{b^2-4(k_f e_t/k_r p_t)(1-k_f e_t/k_r p_t)(K_2(1+\alpha)/m_t)}}{2(k_f e_t/k_r p_t - 1)}, & \frac{k_f e_t}{k_r p_t} \neq 1 \\ \frac{1}{1+K_1(1+\lambda)/K_2(1+\alpha)}, & \frac{k_f e_t}{k_r p_t} = 1 \end{cases} \qquad \text{[S1.17]}$$

where, $b = -(k_f e_t/k_r p_t - 1) + (K_2(1+\alpha)/m_t)(k_f e_t/k_r p_t) + K_1(1+\lambda)/m_t$. Since this expression is same as that in Eq. (7) in the main text, at steady-state, the concentration of phosphorylated form of the substrate.

S1.2. Dose-response curve transition from Signal-Transducing to Hyperbolic regime induced by substrate retroactivity.

We illustrate here the impact of substrate retroactivity in inducing transition in the nature of operating regime from Signal-transducing (ST) to Hyperbolic (H). In Fig S1.1, we show that $\bar{m}_p(9{,}2000)$ in ST regime transitions into H regime when $\lambda = 446$ leading to $\bar{K}_1 = 4023$ while maintaining $\alpha = 0$.

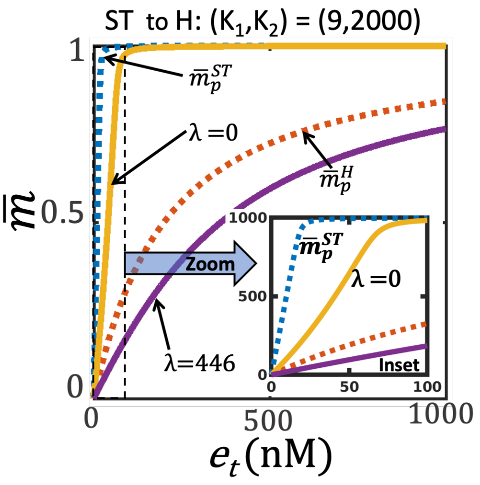

Figure S1.1: Transition of dose-response curve from ST to H. Inset: Zoom in of the dose-response curves. For ease of comparison, the nominal profiles in Figs 2A and 2B are included.

S1.3. Minimum retroactivity strength required to transition from one regime to another. In order to find the minimum retroactivity strength needed to permit a regime transition, we consider each of the five possible transitions achieved by increasing the load λ or α. We demonstrate by considering the case of ST to H transition. For every $(\bar{K}_1(\lambda = 0), \bar{K}_2(\alpha = 0))$ on the ST regime boundary, we estimate λ_{min}, the minimum load on M needed to proportionally scale $\bar{K}_1(\lambda_{min})$, while keeping $\bar{K}_2(\alpha = 0)$ constant, to cause the dose-response curve (solution of Eq. 5 (main text)) to belong to the H regime as per the condition in Eq. 8 (main text). Since this minimum load is sensitive to the chosen $\bar{K}_2(\alpha = 0)$, we find λ_{min} for all $\bar{K}_2(\alpha = 0)$ considered along the ST regime boundary. This procedure is repeated the remaining four regime transitions. The sensitivity to the minimum load λ_{min} or α_{min} due to $\bar{K}_2(\alpha = 0)$ or $\bar{K}_1(\lambda = 0)$ for the considered five transitions are presented in Fig S1.2. The smallest retroactivity strength across all five cases is 0.3 (Fig. S1.2A)

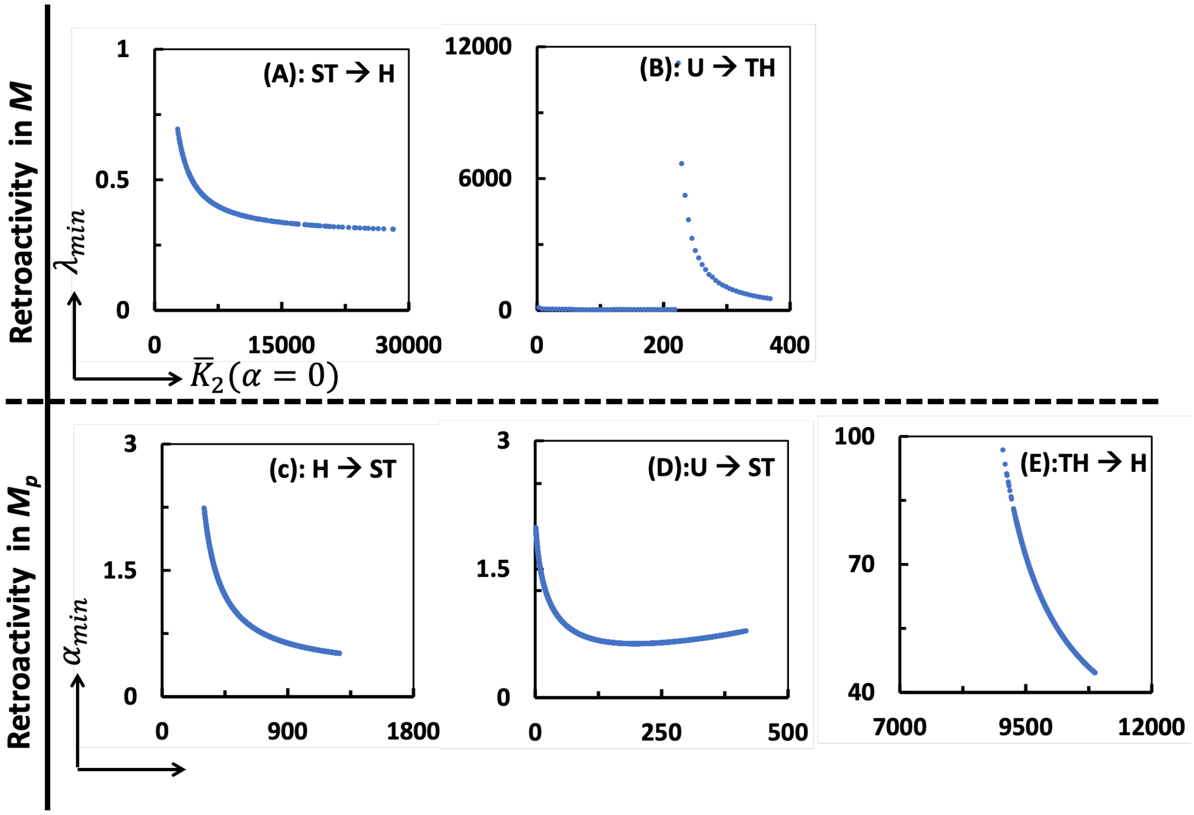

Figure S1.2 Effect of $\bar{K}_2(\alpha = 0)$ on λ_{min} for the transitions (A) ST to H and (B) U to TH, and of $\bar{K}_1(\lambda = 0)$ on α_{min} for the transitions (C) H to ST, (D) U to ST, and (E) TH to H.

S1.4. Effect of retroactivity strength on EC50 during different regime transitions

Changing the retroactivity strength in the substrate modulates the EC50 of the dose-response curve. In Fig. S1.3, we show the variation of EC50, calculated using Eq. 11 (main text) upon changing the retroactivity strength for the five transitions considered in Section 3.3 (main text).

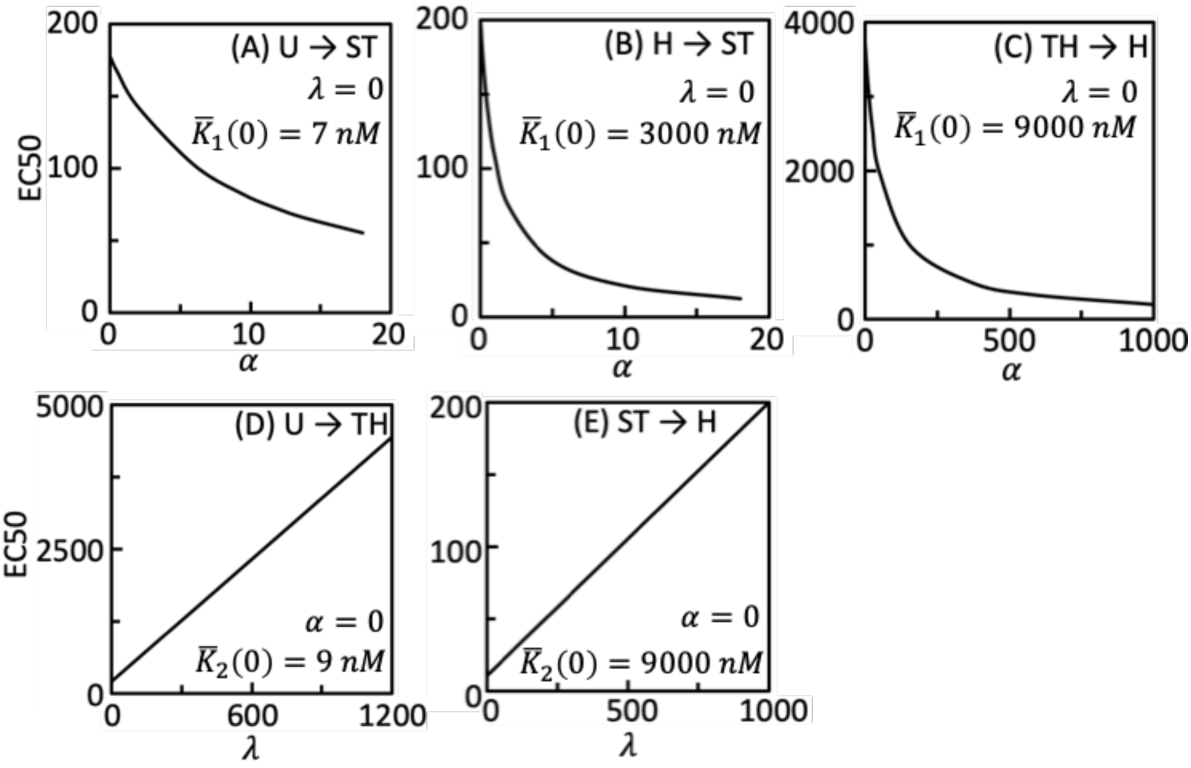

Figure S1.3: Effect of retroactivity strength on EC50 during regime transition for (a) U to TH, (b) U to ST, (c) H to ST, (d) ST to H, (e) TH to H. EC50 is estimated using Eq. 11 (main text).

S1.5. Sensitivity and rate balance analysis to investigate retroactivity induced ST to H and TH to H regime transitions

The retroactivity induced regime transition for ST to H (Fig S1.4) and TH to H (Fig S1.5) are investigated with the help of sensitivity of load (λ *or* α) to enzyme concentration e_t and rate balance analysis for different loads. The dose-response curves corresponding the retroctivity strengths considered here are in Fig. 6 (main text).

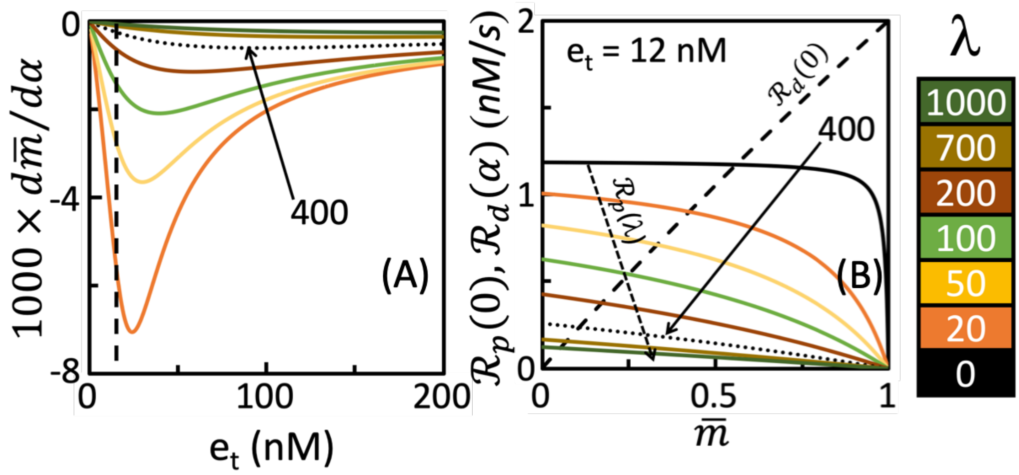

Figure S1.4: Sensitivity (A) and rate balance (B) plot consisting of the locus of $R_p(e_t = 12\,nM, \lambda, \bar{m})$ and $R_d(\alpha = 0\,, \bar{m})$ for ST to H regime transition. Colorbar shows the retroactivity strength for different curves in (A) and (B). Dotted line indicates the sensitivity in (A) and R_d curve in (B) for the retroactivity strength of 400 at the transition. Sensitivity is estimated using Eq. (9), main text.

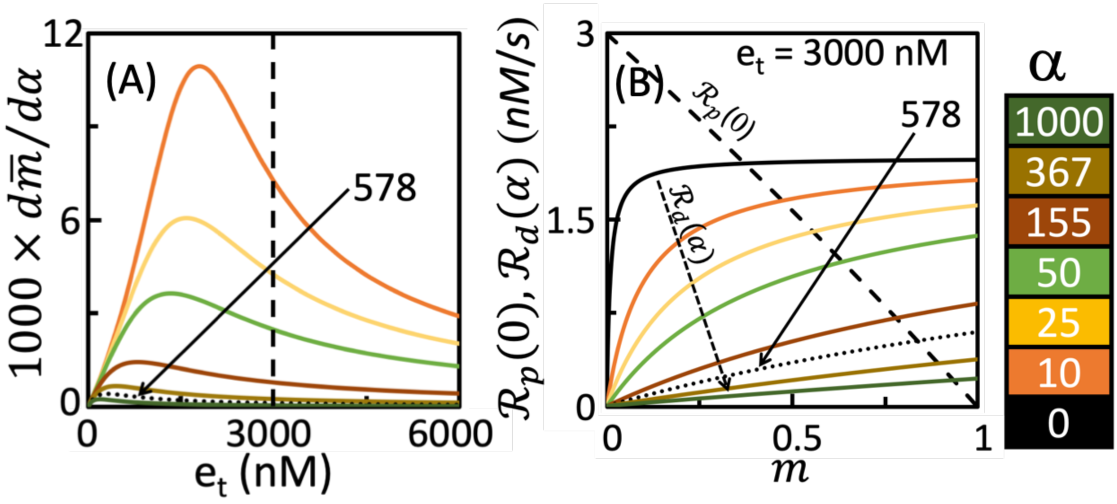

Figure S1.5: (A) Sensitivity of the phosphorylated substrate concentration with respect to the retroactivity strength and (B) rate balance plot consisting of the locus of $R_p(\lambda = 0, \bar{m})$ and $R_d(e_t = 3000\,nM, \alpha, \bar{m})$ for TH to H regime transition. Colorbar shows the retroactivity

strength for different curves in (A) and (B). Dotted line indicates the sensitivity in (A) and R_p curve in (B) for the retroactivity strength of 578 at the transition. Sensitivity is estimated using Eq. (10), main text.
