# Supplementary figures and images for: Retroactivity induced operating regime transition in an enzymatic futile cycle
Source: PLoS One. 2021 Apr 30;16(4):e0250830. doi: 10.1371/journal.pone.0250830 (PMC8087108; doi:10.1371/journal.pone.0250830)

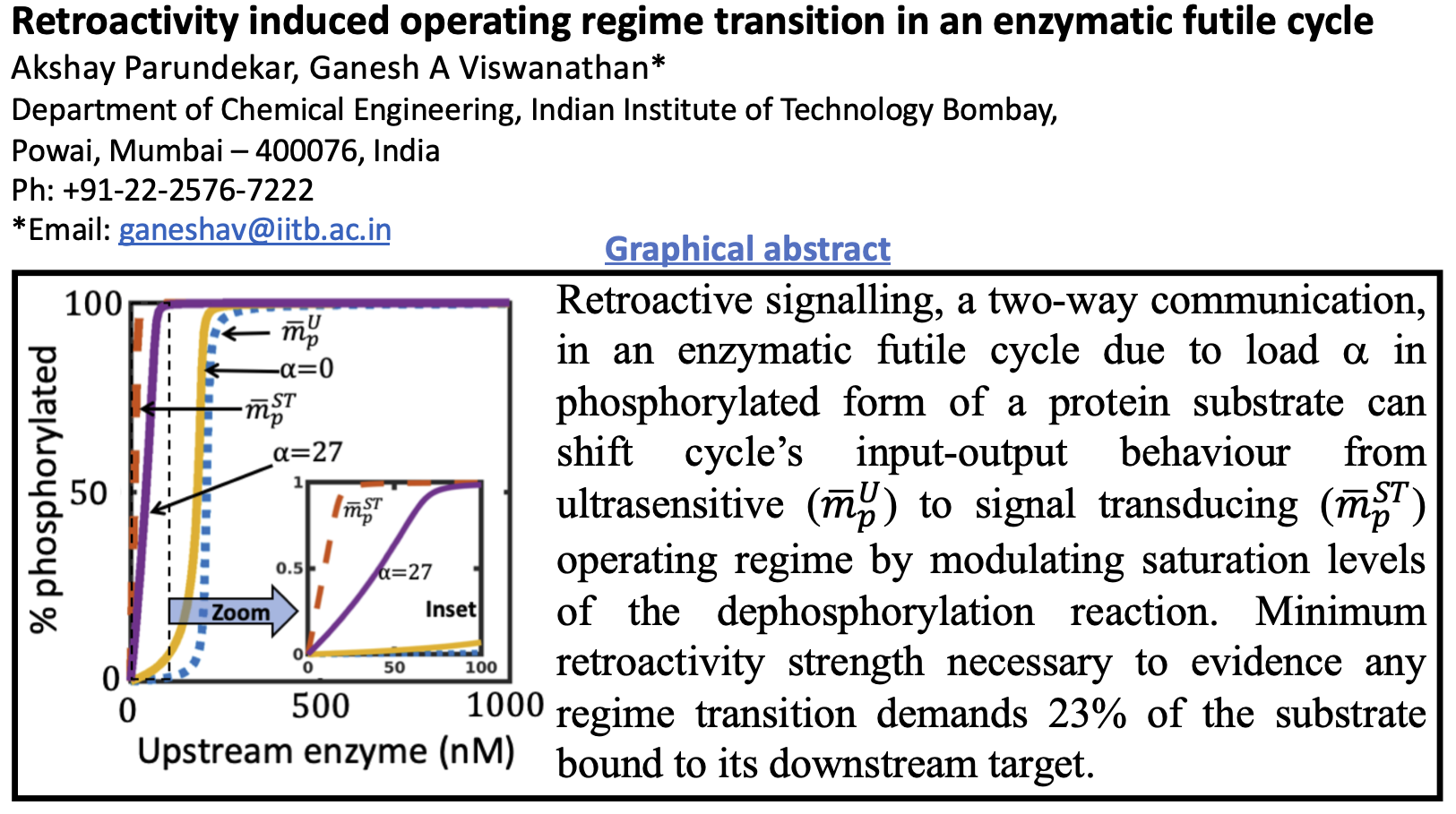

Supplement: S1 Graphical abstract — (TIFF) [file pone.0250830.s002.tiff]
