# Supplementary material for: Retroactivity induced operating regime transition in an enzymatic futile cycle
Source: PLoS One. 2021 Apr 30;16(4):e0250830. doi: 10.1371/journal.pone.0250830 (PMC8087108; doi:10.1371/journal.pone.0250830)
Supplement: S1 Appendix — (DOCX) [file pone.0250830.s003.docx]

**S1 Appendix. Derivation of the mathematical kinetic model**

The dynamics of the complexes formed due to the sequestration reactions (Eqs 3 and 4) is captured by

$\frac{dms_{1}}{dt}=k_{on,1}m^{u}.s_{1}-k_{off,1}ms_{1}$ [AI.1]

$\frac{dm_{p}s_{2}}{dt}=k_{on,2}m_{p}^{u}.s_{2}-k_{off,2}m_{p}s_{2}$ [AI.2]

where, the $ms_{1}$ and $m_{p}s_{2}$ are the concentrations of species $MS_{1}$ and $M_{p}S_{2}$, respectively. In Eqs AI.1 and AI.2, $m^{u}$ and $m_{p}^{u}$, respectively are concentrations of *M* and *M_p_* not bound to their downstream targets. $s_{1}$ and $s_{2}$ are the concentrations of the downstream targets $S_{1}$ and $S_{2}$, respectively. We assume same binding constant values for the two sequestration events, that is$, k_{on,1}=k_{on,2}=k_{on}$ and $k_{off,1}=k_{off,2}=k_{off}$ [24]. The dynamics of the complexes formed due to substrates binding to the corresponding enzymes (Eqs. 1 and 2) are given by

$\frac{d em}{dt}=k_{1}e.m^{u}-\left( k_{-1}+k_{f} \right)em$ [AI.3]

$\frac{d pm_{p}}{dt}=k_{2}p.m_{p}^{u}-\left( k_{-2}+k_{r} \right)pm_{p}$ [AI.4]

where, $em$ and $pm_{p}$ are the concentration of species $EM$ and $PM_{p}$, respectively. As has been assumed in Goldbeter and Koshland [19] and in Ventura et al. [24], ignoring the contributions of the concentrations of $EM$ and $PM_{p}$, we assume that

$m=m^{u}+ms_{1}$ [AI.5]

and

$m_{p}=m_{p}^{u}+m_{p}s_{1}$ [AI.6]

where, $m$ and $m_{p}$ are the total unphosphorylated and phosphorylated substrates, respectively. Further, incorporating the conservation relations for total enzyme concentrations $e_{t}=e+em$ and $p_{t}=p+pm_{p}$ along with Eqs [AI.5] and [AI.6], Eqs [AI.3] and [AI.4] are re-written as

$\frac{d em}{dt}=k_{1}(e_{t}-em).(m-ms_{1})-\left( k_{-1}+k_{f} \right)em$ [AI.7]

$\frac{d pm_{p}}{dt}=k_{2}(p_{t}-pm_{p}).(m_{p}-m_{p}s_{2})-\left( k_{-2}+k_{r} \right)pm_{p}$ [AI.8]

The dynamics of phosphorylated substrate $M_{p}$ is captured by

$\frac{dm_{p}}{dt}=\frac{d}{dt}\left( m_{p}^{u}+m_{p}s_{2} \right)=k_{f}em+k_{-2}pm_{p}-k_{2}(m_{p}-m_{p}s_{2})(p_{t}-pm_{p})$ [AI.9]

Equations [AI.1], [AI.2], [AI.7], [AI.8], and [AI.9] constitute the full model capturing the dynamics of the futile cycle with retroactivity (Fig. 1).

Next, we introduce QSSA for the complexes by setting the lhs of Eqs [AI.1], [AI.2], [AI.7], and [AI.8] to zero. By using Eqs [AI.5] and [AI.6], and rearranging after setting the lhs to zero, Eqs [AI.1] and [AI.2], respectively leads to $ms_{1}=m\lambda/(1+\lambda)$ and $m_{p}s_{2}=m_{p}\alpha/(1+\alpha)$, where $\lambda=s_{1}/K_{d}=s_{1}/(k_{off}/k_{on})$ and $\alpha=s_{2}/K_{d}=s_{2}/(k_{off}/k_{on})$. Next, Eqs [AI.7] and [AI.8], when lhs set to zero, can be rearranged to get

$em=\frac{k_{1}(m-ms_{1})e_{t}}{k_{1}(m-ms_{1})+(k_{-1}+k_{f})}=\frac{me_{t}/(1+\lambda)}{m/(1+\lambda)+K_{1}}=\frac{me_{t}}{m+K_{1}(1+\lambda)}$ [AI.10]

and

$pm_{p}=\frac{k_{2}(m_{p}-m_{p}s_{2})p_{t}}{k_{2}(m_{p}-m_{p}s_{2})+(k_{-2}+k_{r})}=\frac{m_{p}p_{t}/(1+\alpha)}{m_{p}/(1+\alpha)+K_{2}}=\frac{m_{p}p_{t}}{m_{p}+K_{2}(1+\alpha)}$ [AI.11]

where, $K_{1}=(k_{-1}+k_{f})/k_{1}$ and $K_{2}=(k_{-2}+k_{r})/k_{2}$. Setting lhs of Eq. [AI.8] also leads to $k_{2}\left( p_{t}-pm_{p} \right).\left( m_{p}-m_{p}s_{2} \right)=\left( k_{-2}+k_{r} \right)pm_{p}$, substituting which into Eq. [AI.9] results in

$\frac{dm_{p}}{dt}=k_{f}em+k_{-2}pm_{p}-\left( k_{-2}+k_{r} \right)pm_{p}=k_{f}em-k_{r}pm_{p}$ [AI.12]

Substituting for $em$ and $pm_{p}$ from Eqs [AI.10] and [AI.11], respectively into Eq. [AI.12], the quasi-steady state approximated model capturing the dynamics of the futile cycle with sequestration (Fig. 1) can be written as

$\frac{dm_{p}}{dt}=\frac{k_{f}me_{t}}{K_{1}\left( 1+\lambda\right)+m}-\frac{{k_{r}m}_{p}p_{t}}{K_{2}\left( 1+\alpha\right)+m_{p}}=\frac{k_{f}(m_{t}-m_{p})e_{t}}{K_{1}\left( 1+\lambda\right)+(m_{t}-m_{p})}-\frac{{k_{r}m}_{p}p_{t}}{K_{2}\left( 1+\alpha\right)+m_{p}}$ [AI.13]

where, total substrate concentration $m_{t}=m+m_{p}$.
