# Supplementary material for: Retroactivity induced operating regime transition in an enzymatic futile cycle
Source: PLoS One. 2021 Apr 30;16(4):e0250830. doi: 10.1371/journal.pone.0250830 (PMC8087108; doi:10.1371/journal.pone.0250830)
Supplement: S2 Appendix — (DOCX) [file pone.0250830.s004.docx]

# S2 Appendix. Sensitivity of steady-state level to retroactivity strength

Assuming $\delta=\frac{k_{f}e_{t}}{k_{r}p_{t}}$, the expressions for sensitivity of the steady-state level to retroactivity strength due to sequestration of *M* by a downstream target (Eq. 9) is

$\frac{d\bar{m}}{d\alpha}=K_{2}\frac{d\bar{m}}{d\bar{K}_{2}}=\left\{ \begin{matrix} \frac{K_{2}}{m_{t}}\delta\left[ \frac{1+(\bar{K}_{1}\left( \lambda\right)+{\delta\bar{K}}_{2}\left( \alpha\right)+m_{t}(1-\delta))/{(2m}_{t}(\delta-1))}{\sqrt{\left( 1-\delta+\bar{K}_{1}\left( \lambda\right)/m_{t}+{\delta\bar{K}}_{2}\left( \alpha\right)/m_{t} \right)^{2}-4(1-\delta{)\delta\bar{K}}_{2}\left( \alpha\right)/m_{t}}}+\frac{1}{2(1-\delta)} \right], \delta\neq1 \\ \frac{K_{2}\bar{K}_{1}\left( \lambda\right)/\left( \bar{K}_{2}\left( \alpha\right) \right)^{2}}{\left[ 1+{\bar{K}_{1}\left( \lambda\right)}/{\bar{K}_{2}\left( \alpha\right)} \right]^{2}}, \delta=1 \end{matrix} \right.$ [AII.1]

and due to sequestration of *M_p_* by its downstream target (Eq. 10) is given by

$\frac{d\bar{m}}{d\lambda}=K_{1}\frac{d\bar{m}}{d\bar{K}_{1}}=\left\{ \begin{matrix} -\frac{K_{1}}{m_{t}}\left[ \frac{(\bar{K}_{1}\left( \lambda\right)+\delta\bar{K}_{2}\left( \alpha\right)+m_{t}(1-\delta))/{(2m}_{t}(1-\delta))}{\sqrt{\left( 1-\delta+\bar{K}_{1}\left( \lambda\right)/m_{t}+{\delta\bar{K}}_{2}\left( \alpha\right)/m_{t} \right)^{2}-4(1-\delta{)\delta\bar{K}}_{2}\left( \alpha\right)/m_{t}}}+\frac{1}{2(\delta-1)} \right], \delta\neq1 \\ \frac{-K_{1}/\bar{K}_{2}\left( \alpha\right)}{\left[ 1+{\bar{K}_{1}\left( \lambda\right)}/{\bar{K}_{2}\left( \alpha\right)} \right]^{2}}, \delta=1 \end{matrix} \right.$ [AII.2]

where, $\bar{K}_{1}=K_{1}(1+\lambda)$and $\bar{K}_{2}=K_{2}(1+\alpha)$. Definition of all other quantities are as that in Eq. 5.
