# Supplementary material for: Retroactivity induced operating regime transition in an enzymatic futile cycle
Source: PLoS One. 2021 Apr 30;16(4):e0250830. doi: 10.1371/journal.pone.0250830 (PMC8087108; doi:10.1371/journal.pone.0250830)
Supplement: Attachment — Submitted filename: Issues derivation of equation 5.pdf [file pone.0250830.s005.pdf]

To follow the authors' derivation of equation 5, I started with the following mass-action kinetic scheme which is based on the information provided in the text.

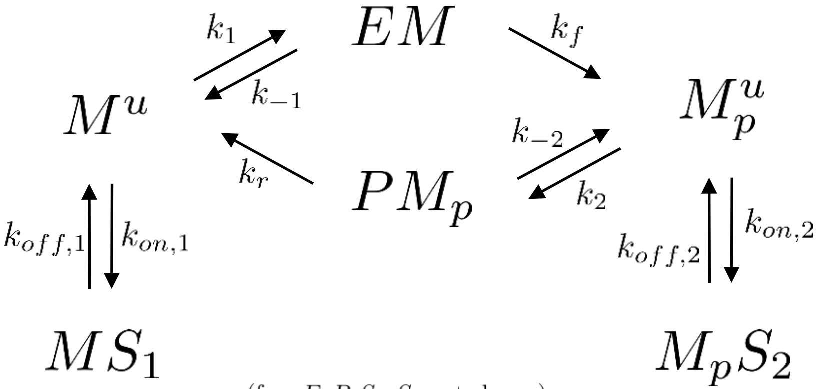

(free E, P, S_1, S_2 not shown)

From this scheme, we can infer five conservation relationships, three of which are as given in appendix I, p.17, lines 402-403:

$$m_t = m_p + m + em + pm_p, \tag{1}$$

$$\text{where } m_p = m_p^u + m_p s_2, \tag{2}$$

$$\text{and } m = m^u + ms_1 \tag{3}$$

$$e_t = e + em \tag{4}$$

$$p_t = p + pm_p \tag{5}$$

The dynamics of the complexes EM and PM_p in the above scheme are described, as given correctly in appendix I, p. 17, lines 405-406, by the equations:

$$\frac{d}{dt}em = k_1 \cdot e \cdot m^u - (k_{-1} + k_f) \cdot em \tag{6}$$

$$\frac{d}{dt}pm_p = k_2 \cdot p \cdot m_p^u - (k_{-2} + k_r) \cdot pm_p \tag{7}$$

For the QSSA we assume that the dynamics complexes EM and PM_p are quasi at steady state compared to the other species in the system, i.e. that $\frac{d}{dt}em = 0$ and $\frac{d}{dt}pm_p = 0$. We substitute into the LHS of equation (6) and solve for em:

$$0 = k_1 \cdot e \cdot m^u - (k_{-1} + k_f) \cdot em \tag{8}$$

$$\stackrel{(5)}{=} k_1 \cdot (e_t - em) \cdot m^u - (k_{-1} + k_f) \cdot em \tag{9}$$

$$= m^u \cdot e_t - em \cdot (m^u + \frac{k_{-1} + k_f}{k_1}) \tag{10}$$

$$\Leftrightarrow em = \frac{m^u \cdot e_t}{m^u + \frac{k_{-1}+k_f}{k_1}} \tag{11}$$

The provided defintion of rectroactivity on M is $\lambda = ms_1/m^u$ (appendix I, p.17, line 400), hence:

$$\begin{aligned} &\Rightarrow m = m^u + ms_1 \overset{(def.\lambda)}{=} m^u + m^u \cdot \lambda = m^u \cdot (1+\lambda) \\ &\Leftrightarrow m^u = m \cdot (1+\lambda)^{-1} \end{aligned}$$

Using the definition of the Michalis constant $K_1 = \frac{k_{-1}+k_f}{k_1}$ we now get from (11) to:

$$em = \frac{e_t \cdot m}{(1+\lambda)\cdot K_1 + m} \qquad (12)$$

By the same procedures we can get from (7) to:

$$pm_p = \frac{p_t \cdot m_p}{(1+\alpha)\cdot K_2 + m_p} \qquad (13)$$

So far this is in agreement with the results provided by the authors. Let us now turn to the dynamics of m_p:

$$\frac{d}{dt}m_p \overset{(2)}{=} \frac{d}{dt}(m_p^u + m_p s_2) = \frac{d}{dt}m_p^u + \frac{d}{dt}m_p s_2 \qquad (14)$$

From the mass-action scheme we can identify

$$\frac{d}{dt}m_p^u = k_f \cdot em + k_{-2}\cdot pm_p + k_{off,2}\cdot m_p s_2 - k_2 \cdot p \cdot m_p^u - k_{on,2}\cdot m_p^u \cdot s_2 \qquad (15)$$

$$\frac{d}{dt}m_p s_2 = k_{on,2}\cdot m_p^u \cdot s_2 - k_{off,2}\cdot m_p s_2 \qquad (16)$$

Substitution of (15) and (16) into (14) yields:

$$\begin{aligned} \frac{d}{dt}m_p =& k_f \cdot em + k_{-2}\cdot pm_p + \cancel{k_{off,2}\cdot m_p s_2} - k_2 \cdot p \cdot m_p^u - \cancel{k_{on,2}\cdot m_p^u \cdot s_2} \\ &+ \cancel{k_{on,2}\cdot m_p^u \cdot s_2} - \cancel{k_{off,2}\cdot m_p s_2} \\ =& k_f \cdot em + k_{-2}\cdot pm_p - k_2 \cdot p \cdot m_p^u \qquad (17) \end{aligned}$$

The correct dynamics of m_p as described by equation (17) are different from the dynamics described by equation [AI.5] provided by the authors on p.17 line 413 and it is easy to see that substituting (12) and (13) into (17) and dividing by m_t as defined in (1) does NOT lead to equation [5] on p.5 in the manuscript.

As an alternative solution, one could lump the complexes into the defintions of m and m_p:

$$m_p \overset{def}{=} m_p^u + m_p s_2 + pm_p \qquad (2)'$$
$$m \overset{def}{=} m^u + ms_1 + em \qquad (3)'$$

The dynamics of m_p are then given by:

$$\begin{aligned}\frac{d}{dt}m_p &\overset{(2)'}{=} \frac{d}{dt}(m_p^u + m_p s_2 + pm_p) = (\frac{d}{dt}m_p^u + \frac{d}{dt}m_p s_2) + \frac{d}{dt}pm_p \\ &= k_f \cdot em + k_{-2} \cdot pm_p - k_2 \cdot p \cdot m_p^u + \frac{d}{dt}pm_p \\ &\overset{(7)}{=} k_f \cdot em + k_{-2} \cdot pm_p - k_2 \cdot p \cdot m_p^u + k_2 \cdot p \cdot m_p^u - (k_{-2} + k_r) \cdot pm_p \\ &= k_f \cdot em + k_r \cdot pm_p \qquad (18)\end{aligned}$$

(18) now has the same form as [AI.5]. Keeping the definitions of K_1 and K_2 and putting (11) and the equivalent for pm_p into (18) yields:

$$\frac{d}{dt}m_p = \frac{k_f \cdot m^u \cdot e_t}{m^u + K_1} - \frac{k_r \cdot m_p^u \cdot p_t}{m_p^u + K_2} \qquad (19)$$

However, from the definition of λ and (2)' we get:

$$m^u = \frac{m - em}{1 + \lambda},$$

so substitution of m^u in (19) would unfortunately reintroduce eliminated complex em. For m_p^u the analogous problem with pm_p. If one defines another pair composite variable similar to the old ones in (2) and (3) e.g. $m^{\backslash em} \overset{def}{=} m^u + ms_1$ and $m_p^{\backslash pm_p} \overset{def}{=} m_p^u + m_p s_2$, the definition of λ and α yields the desired form of (19):

$$\frac{d}{dt}m_p = \frac{k_f \cdot m^{\backslash em} \cdot e_t}{m^{\backslash em} + (1 + \lambda) \cdot K_1} - \frac{k_r \cdot m_p^{\backslash pm_p} \cdot p_t}{m_p^{\backslash pm_p} + (1 + \alpha) \cdot K_2} \qquad (20)$$

Division by m_t would result in the same form as equation [5] from the authors' manuscript.
